# Genome-Wide Identification and Characterization of the UBP Gene Family in Moso Bamboo (*Phyllostachys edulis*)

**DOI:** 10.3390/ijms20174309

**Published:** 2019-09-03

**Authors:** Ruihua Wu, Yanrong Shi, Qian Zhang, Wenqing Zheng, Shaoliang Chen, Liang Du, Cunfu Lu

**Affiliations:** 1 Beijing Advanced Innovation Center for Tree Breeding by Molecular Design, Beijing Forestry University, Beijing 100083, China; 2 College of Biological Sciences and Technology, Beijing Forestry University, Beijing 100083, China

**Keywords:** moso bamboo, UBP genes, phylogenetic analysis, conserved motif, expression patterns

## Abstract

The largest group of deubiquitinases—ubiquitin-specific proteases (UBPs)—perform extensive and significant roles in plants, including the regulation of development and stress responses. A comprehensive analysis of UBP genes has been performed in *Arabidopsis thaliana*, but no systematic study has been conducted in moso bamboo (*Phyllostachys edulis*). In this study, the genome-wide identification, classification, gene, protein, promoter region characterization, divergence time, and expression pattern analyses of the UBPs in moso bamboo were conducted. In total, 48 putative UBP genes were identified in moso bamboo, which were divided into 14 distinct subfamilies in accordance with a comparative phylogenetic analysis using 132 full-length protein sequences, including 48, 27, 25, and 32 sequences from moso bamboo, *A. thaliana*, rice (*Oryza sativa*), and purple false brome (*Brachypodium distachyon*), respectively. Analyses of the evolutionary patterns and divergence levels revealed that the PeUBP genes experienced a duplication event approximately 15 million years ago and that the divergence between PeUBP and OsUBP occurred approximately 27 million years ago. Additionally, several PeUBP members were significantly upregulated under abscisic acid, methyl jasmonate, and salicylic acid treatments, indicating their potential roles in abiotic stress responses in plants.

## 1. Introduction

Post-translational modifications (PTMs) of target proteins control many cellular processes [1,2], and ubiquitination is involved in many physiological events including DNA repair, cell-cycle control, abiotic or biotic stress tolerance, immune responses, endocytosis, and vesicle trafficking [3,4]. Ubiquitination, the covalent attachment of the small protein modifier ubiquitin (Ub) to a substrate protein is catalyzed by three ordered steps conducted by E1 (Ub activating enzyme), E2 (Ub conjugating enzyme), and E3 (Ub ligase), respectively [5,6,7]. Meanwhile, ubiquitination can be reversed by an opposite process, called deubiquitination, which removes Ub from target proteins and cooperates with Ub to regulate the ubiquitination levels of target proteins [4,8]. To date, deubiquitination has been found to be governed by deubiquitinases/deubiquitinating enzymes (DUBs), which generally affect the activities, stabilities, and fates of the target proteins [9,10].

Among the many types of DUBs, the highly conserved ubiquitin-specific proteases (UBPs) form the largest subfamily in plants. UBP proteins contain an Ub carboxyl-terminal hydrolase (UCH) domain with two similar triads of catalytic residues, each containing two short but highly conserved cysteine (Cys) and histidine (His) boxes, which are key parts of the catalytic sites (Cys in the cysteine box, and His and Asp/Asn in the histidine box) [11,12]. In addition to the conserved UCH domain, UBP proteins also have additional non-UBP protein motifs such as the MYND-type zinc finger domain (ZnF-MYND) which has been reported to be a protein–protein interaction domain in mammalian cells, the zinc finger domain ZnF-UBPs, the meprin and TRAF homology (MATH) domains, the domain present in Ubiquitin-Specific Proteases (DUSPs), and Ubiquitin-associated (UBA) domains [13,14]. There are 27 putative UBP family members in *Arabidopsis thaliana* classified into 14 sub-groups, and 21 in rice (*Oryza sativa*) classified into nine sub-groups [12,15]. In plants, UBPs have multiple functions in cell proliferation [13,16], endoreplication [17], root hair elongation [18,19], mitochondria morphogenesis [20], deubiquitination of monoubiquitinated-H2A and -H2B [21,22], pollen development and transmission [22], canavanine resistance [12] and abscisic acid (ABA)-mediated resistance to salt and drought stress [23]. 

Moso bamboo (*Phyllostachys edulis*) is a fast-growing non-timber forest product with high ecological, cultural, economic, and social values, and is extensively used as paper, art ware, and food in China and many other countries [24,25]. However, the normal growth and development of moso bamboo are restrained by the progressively deleterious climate and environmental conditions, such as drought, cold, and salt stress [26]. Years ago, a draft genome of moso bamboo was generated [24], which allows research on the gene families present in moso bamboo. Based on *A. thaliana*, the UBP genes are thought to function throughout plant life. Consequently, identifying their functions in moso bamboo could reveal more roles of UBP genes in plants. In this study, we conducted a systematic, comprehensive analysis of the UBP genes in moso bamboo. We identified 48 putative PeUBP genes and analyzed their phylogenetic relationship, gene structure, protein structure, evolutionary divergence, and expression levels in different tissues. We also examined their expression patterns in response to three plant hormones related to abiotic stress. The results will be beneficial in understanding the roles of *UBPs* in moso bamboo and other plants.

## 2. Results

### 2.1. Identification and Characterization of PeUBP Genes in Moso Bamboo

**Figure 1:**
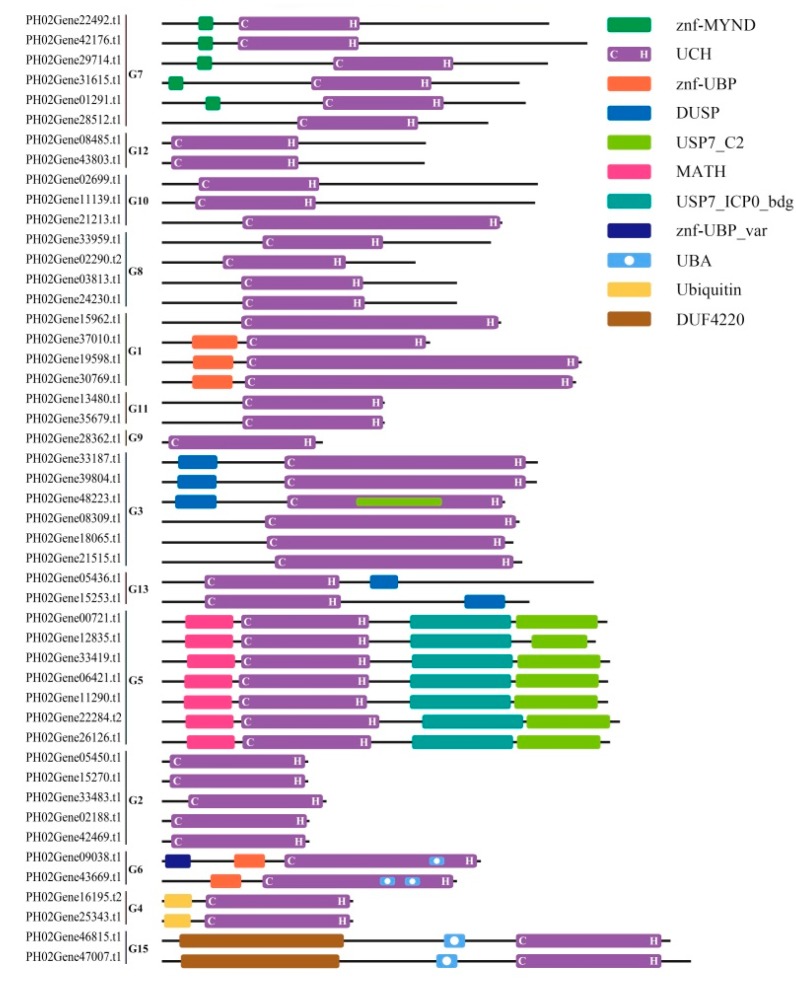
48 PeUBP genes can be divided into 14 groups (G1–G13, and G15) based on the predicted amino acid sequence identities and domain structures. The domains are indicated by different colored boxes.

To carry out a genome-wide identification of the UBP gene family in moso bamboo, Arabidopsis UBP protein sequences were used as queries in searches against the protein databases available in the moso bamboo genome bank (ftp://parrot.genomics.cn/gigadb/pub/10.5524/100001_101000/100498/). We identified 67 PeUBP candidate genes, which were applied to confirm the existence of the conserved UCH domain in UBP proteins using the Pfam database (http://pfam.xfam.org) and the NCBI CD-search program (https://www.ncbi.nlm.nih.gov/Structure/cdd/wrpsb.cgi). After removing sequences that did not satisfy the required conditions, we identified 48 PeUBP genes that exhibited a complete UCH domain with two short but well-conserved motifs specific to UBP proteins (Cys and His) [27]. Like the situation in *A. thaliana*, the members within each subfamily also had the most comparable non-UBP domains, including ZnF-UBPs, MATHs, UBAs, UBQs, and DUSPs domains. Additionally, a bamboo-specific domain, named DUF4220, did not exist in *Arabidopsis* (Figure 1). 

The detailed characteristics of the *PeUBPs*, such as accession number, location, and physicochemical parameters, are given in Table 1. The lengths of the CDSs ranged from 876 to 3915 bp and encoded sequences ranging from 366 to 1159 aa. The molecular weight (MW) varied from 4.20 to 11.58 kDa, and the theoretical isoelectric point (pI) ranged from 4.85 to 9.34. The transmembrane (TM) regions of PeUBP proteins were predicted using TMHMM Server v2.0. The TM regions only existed in four PeUBP members, of which two (PH02Gene46815.t1 and PH02Gene47007.t1), containing three TMs, belonged to the group (G) 15, and two (PH02Gene01291.t1 and PH02Gene29714.t1), with one TM, belonged to G7 (Figure S1).

### 2.2. Phylogenetic Analysis of the PeUBP Genes and Identification of Exon-Intron Structure 

**Figure 2:**
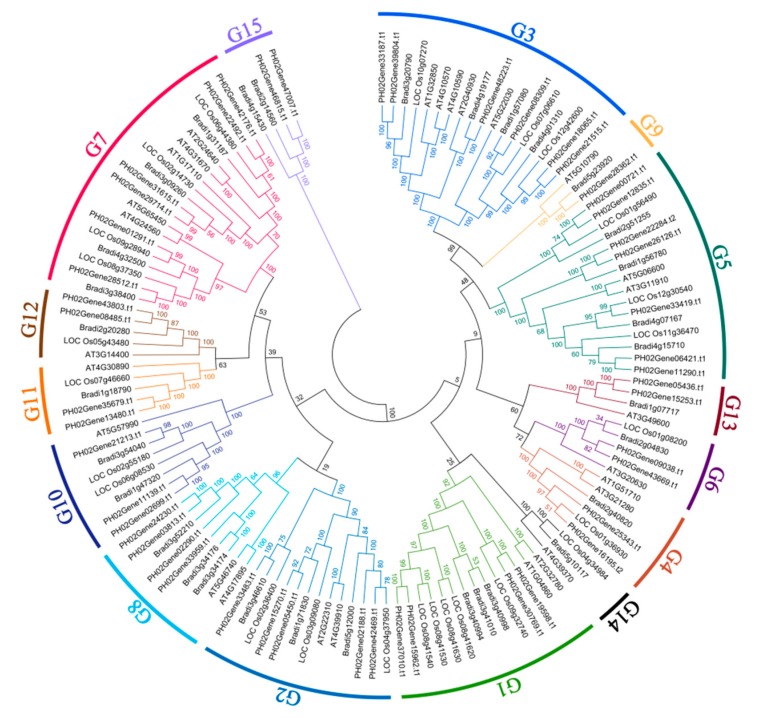
Phylogeny of PeUBP proteins from moso bamboo, *Arabidopsis thaliana*, rice, and *Brachypodium distachyon*. The tree was generated using the Neighbour-joining method with 1000 replicates. The branches of each group are indicated in a specific color.

To analyze the phylogenetic relationship of the UBPs among different species and study the potential functions of PeUBPs, a Neighbor-joining phylogenetic tree was constructed based on the alignments of 132 full-length UBP protein sequences from moso bamboo (48), rice (25) [28], *A. thaliana* (27) [13], and purple false brome (*Brachypodium distachyon*) (29). The detailed characteristics of the UBP genes from *A. thaliana* (Dicotyledonous subfamily), rice (*Oryza sativa*; Poaceae subfamily), and *B. distachyon* (close relationship with moso bamboo; *Monocotyledons*) are listed in supplementary Table S1. In the phylogenetic tree, all sequences were classified into 15 groups, and 48 PeUBPs were distributed into 14 (G1–G13, G15), but not G14. G15 formed a branch with four members from moso bamboo (PH02Gene47007.t1 and PH02Gene45815.t1) and *B. distachyon* (Bradi2g14560 and Bradi4g15430) (Figure 2). In fact, the G15 members had a specific domain (DUF4220) that did not exist in AtUBP and OsUBP proteins. The number of PeUBP members in different groups was uneven. For example, there was none in G14, which had three members, one each from *A. thaliana*, rice, and *B. distachyon*; while in other groups, the number of PeUBPs varied, with seven in G5; four in G1, G7, and G8; two in G4, G6, G11, G12, and G14; and one in both G9 and G10. 

**Figure 3:**
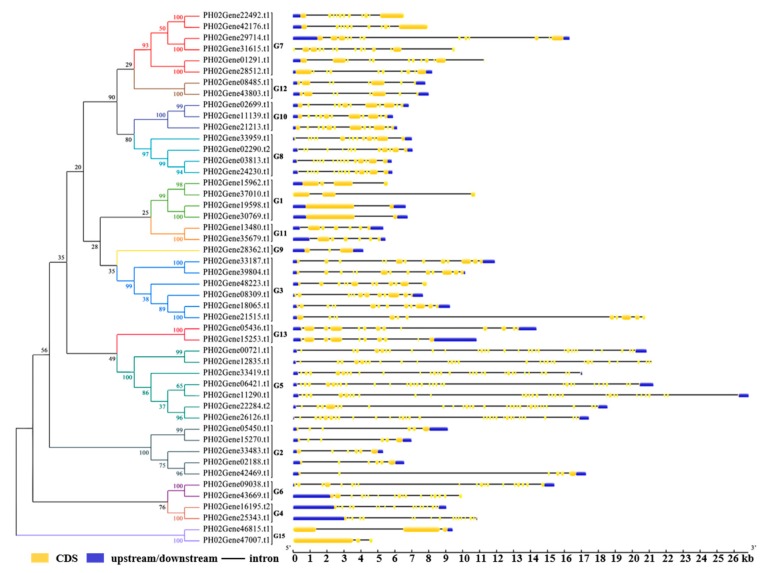
Phylogenetic relationship and gene structures of *PeUBPs* in moso bamboo. Left: Phylogenetic tree of PeUBPs constructed using the Neighbor-joining method based on the results of a sequence alignment. Bootstrap values from 1000 replicates are indicated at each node. The proteins in the tree were divided into 14 distinct subfamilies, and the branches of different subfamilies are marked using different colors. Right: Exons, introns, and untranslated regions (UTRs) are indicated by yellow rectangles, gray lines, and blue rectangles, respectively.

To further analyze the structural diversity of moso bamboo PeUBP genes, a separate phylogenetic tree was constructed only using the full-length UBP protein sequences of moso bamboo. Moso bamboo proteins were also divided into 14 distinct subfamilies, in good agreement with those of the four plant species (Figure 2 and Figure 3). The Gene Structure Display Server (GSDS) online tool was used to identify the exon-intron structure of each predicted PeUBP gene. As shown in Figure 3, the five PeUBP genes (PH02Gene00721.t1, PH02Gene06421.t1, PH02Gene11290.t1, PH02Gene26126.t1, and PH02Gene33419.t1) in G5 contained the largest number of exons (30), and another two genes (PH02Gene12835.t1 and PH02Gene22282.t1) in G5 contained 30 and 31 exons, respectively. In the other 41 PeUBP genes, the exon numbers varied from 2 to 19. Upstream and downstream sequences commonly existed in the PeUBP genomic sequences except for in PH02Gene31615.t1, PH02Gene379010.t1, and PH02Gene47007.t1, which lacked upstream and downstream sequences. Besides, PH02Gene01291.t1, PH02Gene12835.t1, PH02Gene15962.t1, PH02Gene21515.t1, PH02Gene22492.t1, PH02Gene42176.t1, PH02Gene43669.t1, and PH02Gene48223.t1 have only upstream sequences, while PH02Gene46815 has only a downstream sequence. The intron/exon structure of sister gene pairs had both conservative and differential regions. For example, most gene pairs varied greatly in their structural organization and the numbers of intron-exons, except for six gene pairs (PH02Gene02188.t1/PH02Gene42469.t1, PH02Gene03813.t1/PH02Gene379010.t1, PH02Gene05450.t1/PH02Gene15270.t1, PH02Gene08485.t1/PH02Gene43803.t1, PH02Gene13480.t1/PH02Gene35679.t1, and PH02Gene19598.t1/PH02Gene30769.t1), which were shown to have the same intron-exon numbers and intron phase but with variable intron lengths.

### 2.3. Identification of Conserved Sequence Motifs and Determination of Homology Modeling in Moso Bamboo UBP Genes

**Figure 4:**
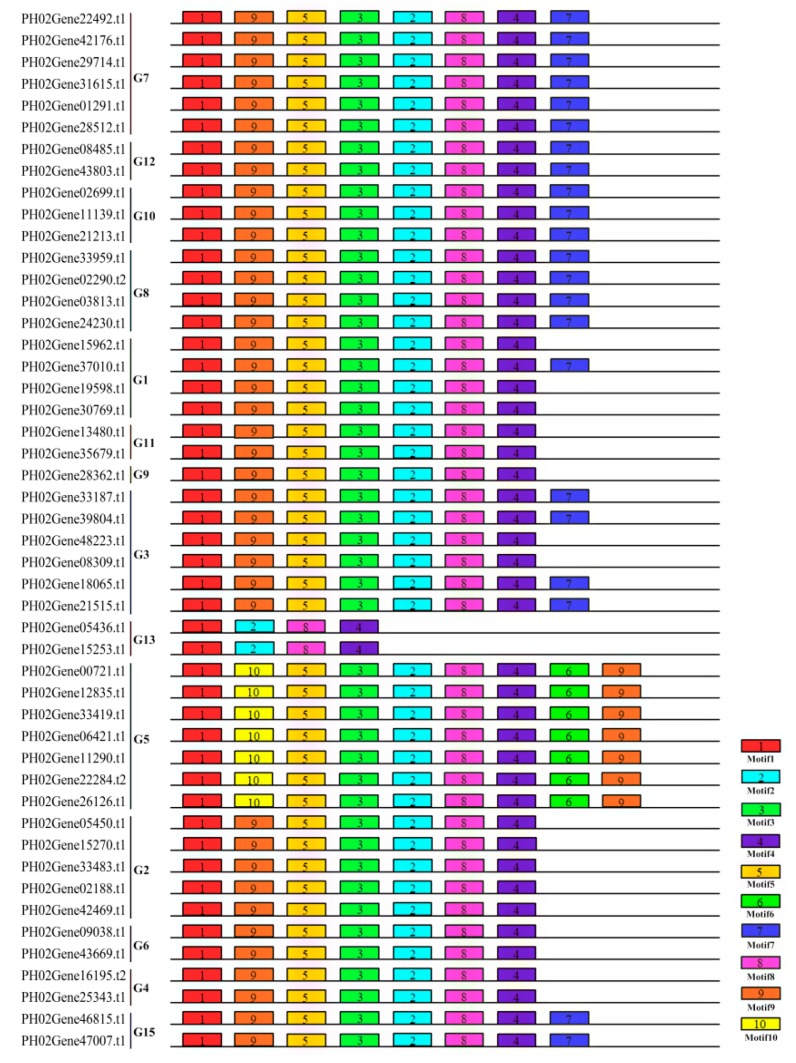
Schematic representation of the ten conserved motifs in PeUBPs. Conserved motifs of the PeUBPs were identified using the online MEME program based on 48 full-length aa sequences. The lengths of the motifs are displayed proportionally. The numbers in boxes (1–10) represent the motif numbers.

To obtain the compositions and diversification of motifs present in the 48 PeUBP proteins, we searched for the conserved motifs using the MEME program (http://meme-suite.org/tools/meme) (Figure 4). A total of ten distinct motifs were identified, and detailed information on the Logo sequences of these ten motifs are provided in Table S2. Members classified into the same groups had similar or identical conserved motifs, indicating functional similarities among these proteins. All the PeUBP proteins were characterized by Motif 1 in the N-terminal UCH domain, and Motifs 4 and 8 in the C-terminal UCH domain, which represented the Cys and His boxes, respectively. With a few exceptions, the motifs in most of the PeUBP proteins had the same order ranking of 1, 9, 5, 3, 2, 8, and 4. In addition, motifs 6 and 10 were exclusively found in G5.

**Figure 5:**
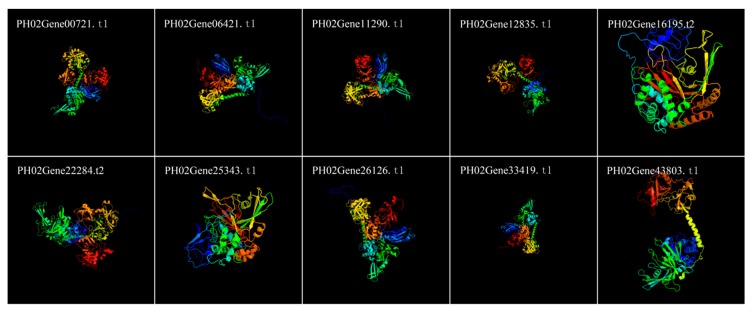
Predicted structures of PeUBP proteins. The structures of 10 PeUBP proteins were predicted with > 90% confidence.

To determine the structures of the PeUBP proteins, we used Phyre2 (http://www.sbg.bio.ic.ac.uk/phyre2/html/page.cgi?id=index) to predict the homology modeling, and we aligned the PeUBP protein sequences using the HMM-HMM search in intensive mode [29]. All of the 48 PeUBP proteins could be confidently modeled. As shown in Figure 5, 10 PeUBPs had 100% of their predicted lengths modeled with > 90% confidence (PH02Gene00721.t1, PH02Gene06421.t1, PH02Gene11290.t1, PH02Gene12835.t1, PH02Gene16195.t2, PH02Gene22284.t2, PH02Gene25343.t1, PH02Gene26126.t1, PH02Gene33419.t1, and PH02Gene43803.t1).

### 2.4. Evolutionary and Divergence Patterns of the UBP Genes in Moso Bamboo, Rice, and B. distachyon

**Figure 6:**
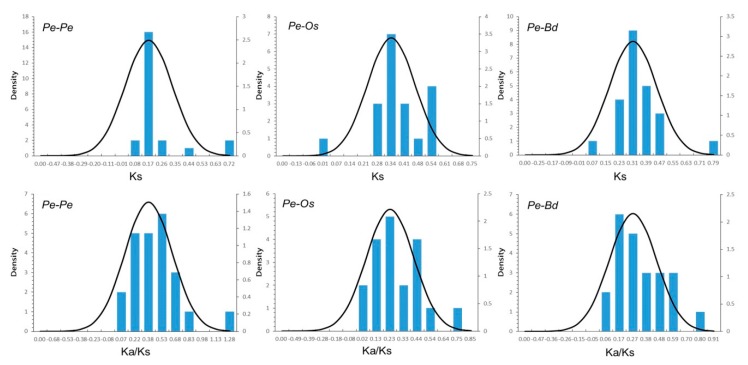
Ks and Ka/Ks value distributions of the PeUBP genes in the genomes of moso bamboo, rice, and *Brachypodium distachyon*, viewed through the frequency distribution of relative Ks and Ka/Ks modes. Distributions of Ks and Ka/Ks values were obtained from paralogous gene pairs in the moso bamboo genome, orthologous gene pairs between moso bamboo and rice, and orthologous gene-pairs between moso bamboo and *B. distachyon*.

To analyze the UBP’s evolutionary and divergence patterns, homologous pairs among moso bamboo, rice, and *B. distachyon* were subjected to BLASTn sequence similarity analyses. 21 putative paralogous (Pe-Pe) in moso bamboo, 18 orthologs (Pe-Os) between moso bamboo and rice, and 22 orthologs (Pe-Bd) between moso bamboo and *B. distachyon* were identified (Table 2). The divergence times of moso bamboo, rice, and *B. distachyon* were evaluated using the formula T = Ks/2λ, and with the Ks value serving as a proxy for time. The relative Ks values of paralogous pairs (Pe-Pe) averaged ~0.2, indicating that PeUBP genes experienced a large-scale duplication event approximately 15 million years ago (MYA). A previous study reported that bamboo underwent whole-genome duplication 7–12 MYA, indicating that the large-scale duplication of the UBP genes occurred earlier [24]. The Ks value distributions of Pe-Os and Pe-Bd orthologous pairs both peaked at approximately 0.35 (Figure 6), indicating that the divergence time of these genes was 27 MYA. A comparison with a previous study revealed that the divergence times between moso bamboo and rice, and moso bamboo and *B. distachyon* were 43–57, 42–52 MYA, respectively, indicating that the UBP genes underwent gene evolution prior to the separation of the two progenitor species. In general, Ka/Ks ratio greater than 1, equal to 1, and less than 1 indicate that a gene has experienced positive, neutral, and negative or stabilizing selection, respectively [30,31]. The Ka/Ks ratios of Pe-Pe, Pe-Os, and Pe-Bd genomes were all less than 1, which suggested that the UBP genes between moso bamboo and rice genomes and moso bamboo and Brachypodium genomes, as well as for the paralogous in the moso bamboo genome, have experienced strongly positive purifying selection.

### 2.5. PeUBP Expression Levels in Different Tissues

**Figure 7:**
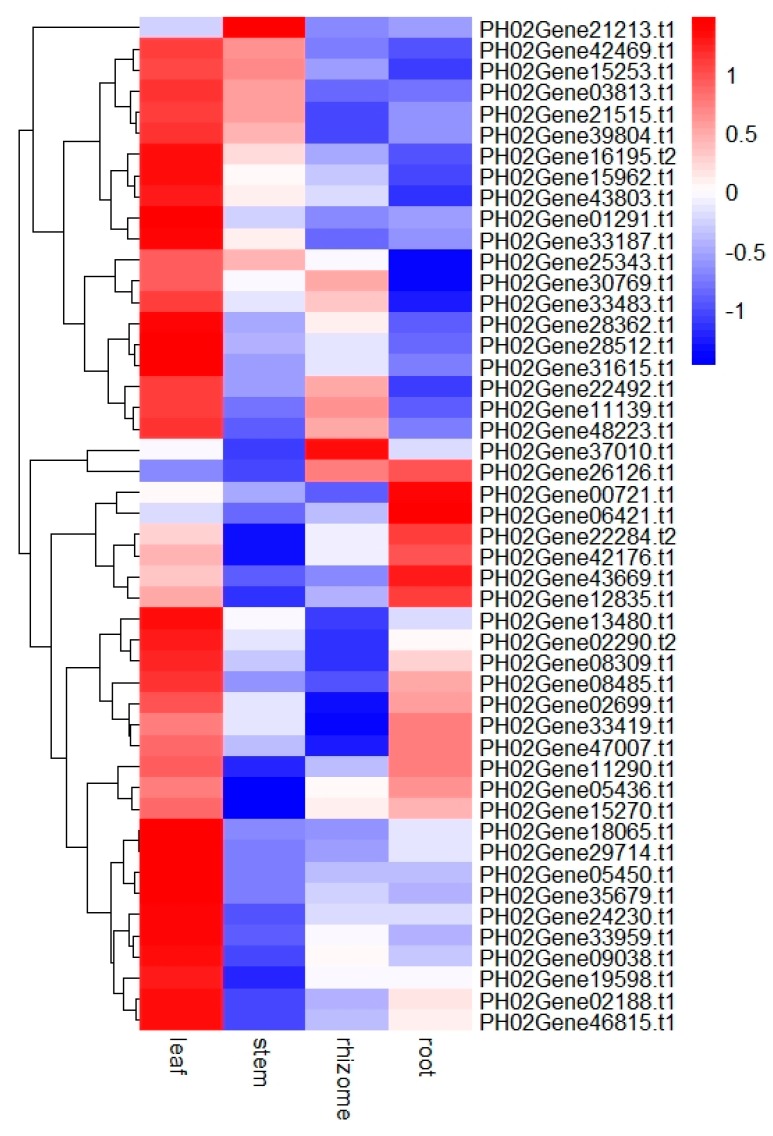
Expression profiles of PeUBP genes in different tissues of moso bamboo. Samples were from leaf, stem, rhizome, and root. The heatmap shows the hierarchical clustering of 48 PeUBP genes across the different tissues analyzed. The color scale presented vertically at the right side of the picture represents log10 expression values; blue and red represents low and high levels of transcript abundance. The mean values were obtained from three biological and three technical replicates.

Tissue-specific gene expression provides vital clues for dissecting gene function. To characterize the expression patterns of PeUBP genes, we analyzed their transcription levels in different tissues of moso bamboo, including leaf, stem, rhizome, and root using quantitative real-time PCR (qRT-PCR) (Table S3). As shown in Figure 7, most PeUBP genes (except for PH02Gene00721.t1, PH02Gene06421.t1, PH02Gene21213.t1, PH02Gene26126.t1, and PH02Gene37010.t1) in the leaf, seven genes (PH02Gene03813.t1, PH02Gene15253.t1, PH02Gene21213.t1, PH02Gene21515.t1, PH02Gene25343.t1, PH02Gene39804.t1, and PH02Gene42469.t1) in the stem, seven genes (PH02Gene11139.t1, PH02Gene22492.t1, PH02Gene26126.t1, PH02Gene30769.t1, PH02Gene33483.t1, PH02Gene37010.t1, and PH02Gene48223.t1) in the rhizome, and 14 genes (PH02Gene00721.t1, PH02Gene02699.t1, PH02Gene05436.t1, PH02Gene06421.t1, PH02Gene08485.t1, PH02Gene11290.t1, PH02Gene12835.t1, PH02Gene15270.t1, PH02Gene22284.t1, PH02Gene26126.t1, PH02Gene33419.t1, PH02Gene42176.t1, PH02Gene43669.t1, and PH02Gene47007.t1) in the root showed high expression levels. In particular, for all the PeUBPs, the expression levels were significantly higher in leaf than in other tissues. We also determined that some genes presented a tissue-specific expression profile. For example, PH02Gene05436.t1, PH02Gene11290.t1, PH02Gene15270.t1, PH02Gene33419.t1, and PH02Gene47007.t1 showed high expression levels in leaves and roots, but low levels in stems and rhizomes. PH02Gene03813.t1, PH02Gene15253.t1, PH02Gene21515.t1, PH02Gene39804.t1, and PH02Gene42469.t1 showed high expression levels in the leaf and stem, but low levels in rhizome and root. PH02Gene11139.t1, PH02Gene22492.t1, PH02Gene48223.t1 showed high expression levels in the leaves and rhizomes, but low levels in stems and roots. Only PH02Gene21213.t1 was highly expressed in the stem, and only PH02Gene37010.t1 was highly expressed in the rhizome. In addition, PH02Gene00721.t1 and PH02Gene06421.t1 showed relatively high expression levels in the root compared with the other three tissues. Above all, PeUBP genes exhibited different expression profiles in different tissues, indicating the multiple biological functions of *PeUBPs* in moso bamboo growth and development.

### 2.6. Expression Profiles of PeUBP Genes under Abscisic Acid (ABA), Methyl Jasmonate (MeJA), and Salicylic Acid (SA) Treatments

**Figure 8:**
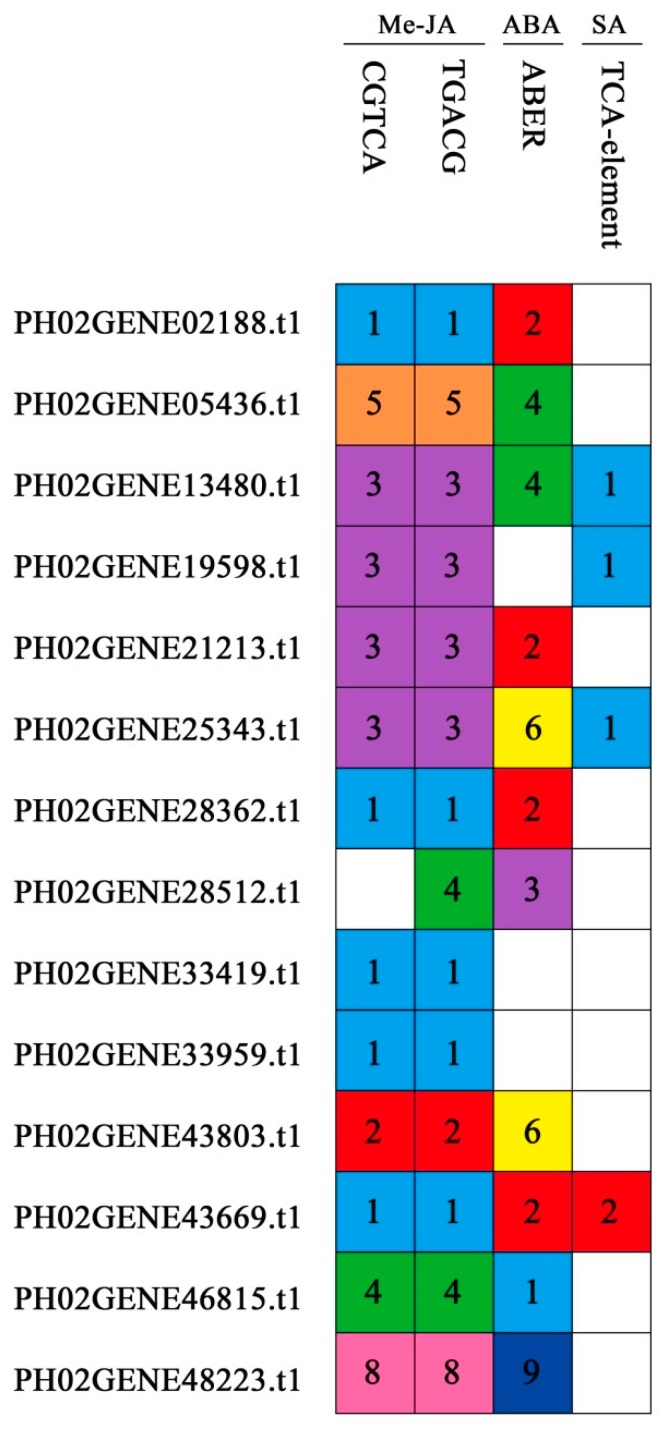
*cis*-Acting elements related to ABA, MeJA, and SA in the promoter regions of PeUBPs. The colored blocks containing numbers represent the numbers of *cis*-element in PeUBPs.

Several *AtUBPs* have been reported to function in the regulation of ABA (*AtUBP24*) [23] and MeJA (*AtUBP12/13*) [32,33,34]; consequently, we hypothesized that the PeUBP genes also had similar functions. We analyzed the promoters of all the PeUBP genes using PlantCARE (http://www. dna.affrc.go.jp/PLACE/) to examine the 2000-bp upstream sequences. Many cis-regulatory elements corresponding to ABA, MeJA, and SA were found, indicating that PeUBPs played roles in plant development and stress responses (Figure 8) [35,36,37,38,39]. 

Then, we performed qRT-PCR to evaluate the dynamic expression levels under ABA, MeJA, and SA treatments, with 14 genes as representatives of each subfamily (PH02Gene02188.t1, PH02Gene05436.t1, PH02Gene13480.t1, PH02Gene19598.t1, PH02Gene21213.t1, PH02Gene25343.t1, PH02Gene28362.t1, PH02Gene28512.t1, PH02Gene33419.t1, PH02Gene33959.t1, PH02Gene43669.t1, PH02Gene43803.t1, PH02Gene46815.t1, and PH02Gene48223.t1). 

**Figure 9:**
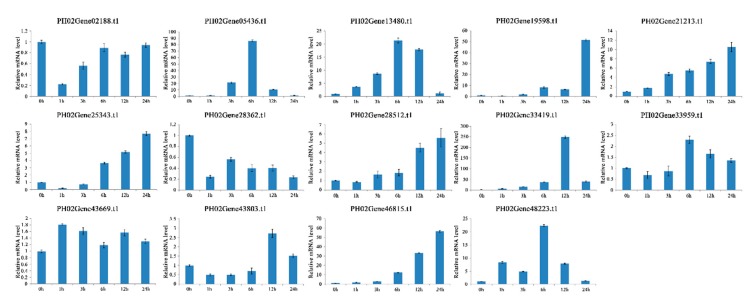
Expression analyses of 14 representative PeUBP genes under MeJA treatment. Sampling occurred 0, 1, 3, 6, 12, and 24 h after treatment, and the relative expression levels were analyzed.

In the MeJA treatment, nine genes (PH02Gene05436.t1, PH02Gene13480.t1, PH02Gene19598.t1, PH02Gene21213.t1, PH02Gene25343.t1, PH02Gene28512.t1, PH02Gene33419.t1, PH02Gene46815.t1, and PH02Gene48223.t1) were distinctly up-regulated. For example, the expression of three genes (PH02Gene05436.t1, PH02Gene 13480.t1, and PH02Gene48223.t1) peaked at 6h, with a gradual decrease at all later time points. The PH02Gene33419.t1 was the most highly expressed (>200-fold that of 0 h) after 12 h of treatment. Five genes (PH02Gene19598.t1, PH02Gene21213.t1, PH02Gene25343.t1, PH02Gene28512.t1, and PH02Gene46815.t1) peaked at 24 h, with a gradual increase over time, except for PH02Gene19598.t1. By contrast, PH02Gene28362.t1 was down-regulated under the MeJA treatment. Additionally, there were five PeUBP genes (PH02Gene02188.t1, PH02Gene28362.t1, PH02Gene33959.t1, PH02Gene43669.t1, and PH02Gene43803.t1) that showed slight (<5-fold that at 0 h) changes in response to MeJA treatment (Figure 9).

**Figure 10:**
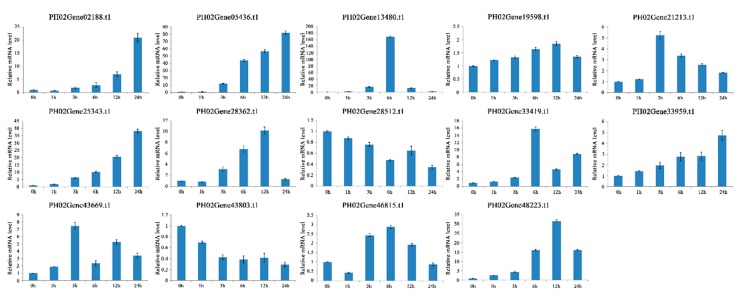
Expression analyses of 14 representative PeUBP genes under ABA treatment. Sampling occurred 0, 1, 3, 6, 12, and 24 h after treatment, and the relative expression levels were analyzed. Untreated sample expression levels = 1. *X*-axes and *Y*-axes represent time points after ABA treatment and relative gene expression values normalized to reference gene *TIP41*, respectively. Bars indicate standard deviations from three biological replicates.

In the ABA treatment, nine genes (PH02Gene02188.t1, PH02Gene05436.t1, PH02Gene13480.t1, PH02Gene21213.t1, PH02Gene25343.t1, PH02Gene28362.t1, PH02Gene33419.t1, PH02Gene43669.t1, and PH02Gene48223.t1) were distinctly up-regulated. For example, two genes (PH02Gene21213.t1 and PH02Gene43669.t1) were gradually up-regulated during the early time points, peaked at 3 h, and decreased during subsequent treatment. Two genes (PH02Gene13480.t1, and PH02Gene33419.t1) were the most highly expressed at 6 h, with a gradual increase during the early time points and a significant decrease during subsequent treatments. Moreover, PH02Gene13480.t1 showed an expression level more than 160-fold higher at 6 h than at 0 h. The expression levels of two genes (PH02Gene 28362.t1 and PH02Gene48223.t1) were highest at 12 h. However, the expression levels of five genes (PH02Gene19598.t1, PH02Gene28512.t1, PH02Gene33959.t1, PH02Gene43803.t1, and PH02Gene46815.t1) changed only slightly (<5 fold that at 0 h) during the 24-h time course (Figure 10). 

**Figure 11:**
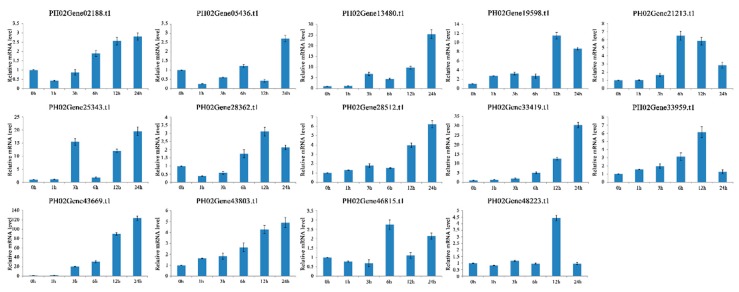
Expression analyses of 14 representative PeUBP genes under SA treatment. Sampling occurred 0, 1, 3, 6, 12, and 24 h after treatment, and the relative expression levels were analyzed. Untreated sample expression levels = 1. *X*-axes and *Y*-axes represent time points after SA treatment and relative gene expression values normalized to reference gene *TIP41*, respectively. Bars indicate standard deviations from three biological replicates.

In the SA treatment, eight genes (PH02Gene13480.t1, PH02Gene19598.t1, PH02Gene21213.t1, PH02Gene25343.t1, PH02Gene28512.t1, PH02Gene33419.t1, PH02Gene33959.t1, and PH02Gene43669.t1) were distinctly up-regulated. For example, PH02Gene21213.t1 exhibited a gradual increase in expression during the early time points, a peak at 6 h, and a gradual decrease at all later time points. The expression levels of two genes (PH02Gene19598.t1 and PH02Gene33959.t1) were highest at 12 h. Five genes (PH02Gene13480.t1, PH02Gene25343.t1, PH02Gene28512.t1, PH02Gene33419.t1, and PH02Gene43669.t1) were the most highly expressed at 24 h, especially PH02Gene43669.t1, with an expression level over 100-fold higher at 24 h than at 0 h. In addition, the expression levels of six genes (PH02Gene01288.t1, PH02Gene05436.t1, PH02Gene28362.t1, PH02Gene43803.t1, PH02Gene46815.t1, and PH02Gene48223.t1) changed only slightly (<5-fold that at 0 h) over the 24-h time course (Figure 11).

In total, four genes (PH02Gene13480.t1, PH02Gene21213.t1, PH02Gene25343.t1, and PH02Gene33419.t1) showed significant changes in response to all three hormone treatments. Additionally, these results were consistent with the putative promoter analysis of PeUBP members that revealed the wide distribution of several ABA-, MeJA-, and SA- responsive cis-elements in these four genes. This implied that a number of PeUBP genes had potential functions in response to ABA, Me-JA, and SA. 

Untreated sample expression levels = 1. X-axes and Y-axes represent time points after MeJA treatment and relative gene expression values normalized to reference gene TIP41, respectively. Bars indicate standard deviations from three biological replicates.

## 3. Discussion

### 3.1. UBPs in Moso Bamboo

The eukaryotic-specific UBP family, which is one of the largest families of DUBs determined to date, plays an essential role in the processes of plant growth and development [40]. According to a previous study, detailed characteristics and functions of UBP genes have been uncovered in *A. thaliana* [7]. However, until now, the UBP family members have not been described in moso bamboo. Here, we identified 48 putative UBP genes in moso bamboo using a genome-wide analysis and compared them with 27 *AtUBPs*, 25 *OsUBPs,* and 32 *BdUBPs*. The greater number of PeUBP genes in moso bamboo among these four species was consistent with a genome duplication event occurring in moso bamboo [24,41]. Based on the phylogenetic analysis, the predicted PeUBP gene family was classified into 14 groups (G1–G13, G15). Among them, G1 to G13 shared the same type of domains as those in the corresponding groups of UBP genes in *A. thaliana*, while PH02Gene47007.t1 and PH02Gene45815.t1 in G15 contained a special DUF4220 domain that only existed in moso bamboo and *B. distachyon*. In addition, there were no orthologs genes in moso bamboo G14, suggesting a divergence among *A. thaliana,* rice, *B. distachyon*, and moso bamboo. 

### 3.2. Divergence of UBPs in Moso Bamboo, Rice, and Brachypodium

Gene duplication events help organisms adapt to variant environments during development and growth, and are also essential for gene evolution and expansion [42,43]. To better explore the macroevolutionary patterns and evolutionary rates in moso bamboo, we analyzed the Ks and Ka models of paralogous genes (Pe-Pe) and orthologous genes (Pe-Os and Pe-Bd) and calculated the Ks and Ka/Ks values for each gene pair. The Ks values indicated that a large-scale duplication event occurred approximately 15 MYA in moso bamboo and that the divergence times for orthologous genes (Pe-Os and Pe-Bd) were both approximate 27 MYA. A previous report showed that a whole-genome duplication event occurred 7–12 MYA in moso bamboo. The divergence time between moso bamboo and rice was 48.6 MYA, while that of moso bamboo and *B. distachyon* was 46.9 MYA [22]. In addition, the Ka/Ks ratio can be used to determine whether selective pressure acts on the protein-coding gene. Here, the Ka/Ks ratios were all less than 1, which indicated that the PeUBP homologous gene pairs have undergone purification selection during the evolutionary process [30,31].

### 3.3. The Potential Functions of UBPs in Development and Stress-Responses in Moso Bamboo

The analysis of UBP gene expression profiles in different tissues contributed to understanding gene functions in moso bamboo growth and development. Here, we verified the expression levels of 48 putative PeUBP genes in different tissues (leaf, stem, rhizome, and root). Most PeUBP genes were highly expressed in leaf, suggesting that *PeUBPs* might be involved in leaf growth and development. However, the high expression level in leaf might result from the faster growth and development, which requires more active metabolism or gene activities. A few PeUBP genes showed tissue-specific expression in moso bamboo. For example, PH02Gene43669.t1—the orthologue of AtUBP14—showed high expression levels in leaf and root, indicating that it plays a role in leaf and root development, similar to *AtUBP14,* which controls leaf size and root hair elongation [44]. PH02Gene00721.t1, PH02Gene06421.t1, PH02Gene12835.t1, PH02Gene26126.t1, PH02Gene33419.t1, and PH02Gene43669.t1, the orthologues of *AtUBP12* and *AtUBP13,* were highly expressed in the root, indicating that these genes might function in moso bamboo root development, coinciding with the functions of *AtUBP12* and *AtUBP13* in Arabidopsis root meristem maintenance and development [33,34].

Tissue-specific and stress-responsive gene expression patterns are largely dependent upon *cis*-elements in the promoter region. Additionally, the *cis*-regulatory elements are also closely related to multiple stimuli-responsive genes [27,40]. In our study, we analyzed the *cis*-elements of PeUBPs and found many MeJA-, ABA-, and SA- responsive sequences in PeUBP promoters, indicating the significant roles of PeUBPs in MeJA-, ABA-, and SA- stress responses [45]. 

The three plant hormones (ABA, MeJA, and SA) have established a good role in plant stress response signaling systems, growth, and development processes. To date, the associations of plant UBP with ABA and MeJA have been uncovered. ABA is produced in dehydrated plant tissues and mature seeds under water deficiency and regulates the expression levels of many genes that take part in drought tolerance [46]. A report [23] showed that AtUBP24 functions upstream of ABI2, and negatively regulates phosphatase activity of PP2C, demonstrating the regulatory role of AtUBP24 in response to ABA in plants. As the homologous gene of *AtUBP24* in moso bamboo, the PH02Gene13480.t1 has the same protein structure and conserved domains. Its expression pattern is different from *AtUBP24* [23]. However, the PH02Gene13480.t1 showed high ABA sensitivity in our experiments, implying that PH02Gene13480.t1 possibly has a similar role in the ABA-stress response. As a possible air signaling molecule, MeJA plays an important role in regulating communication within and between plants and in regulating plant defense responses, including antioxidant systems [47]. AtUBP12/AtUBP13 positively regulate MYC2 levels in MeJA responses [48] and are involved in plant immunity, circadian clock, and root meristem maintenance [32,34]. Their homologous gene in moso bamboo, PH02Gene33419.t1, had the same conserved UCH and MATH domains and showed high expression levels in the young leaf and root, similarly to *AtUBP12/AtUBP13* [48]. In addition, the PH02Gene33419.t1 expression level rises sharply after the MeJA treatment, indicating that PH02Gene33419.t1 may play an important role in MeJA responses, similarly to AtUBP12 and AtUBP13 [48]. 

## 4. Materials and Methods 

### 4.1. Identification of Moso Bamboo PeUBP Genes

The UBP protein sequences were originally used as seed sequences to search the Bamboo database (http://parrot.genomics.cn). Then the redundant sequences were removed based on the BLAST results of a ClustalW 2.1 alignment [49], and the putative PeUBP members were confirmed using Pfam (http://pfam.xfam.org/) and the NCBI CD-search program (https://www.ncbi.nlm.nih.gov/Structure/cdd/wrpsb.cgi) [50,51]. The information on the amino acids, CDS lengths, and physicochemical parameters of the PeUBP genes were obtained from the Bamboo database (http://parrot.genomics.cn). By comparing the CDSs and the corresponding genomic DNA sequences of PeUBP genes, we obtained their exon-intron structures from GSDS (http://gsds.cbi.pku.edu.cn/). Bioinformatic analysis of PeUBP genes was performed using ExPASy (http://www.expasy.ch/tools/pi_tool.html) to determine the number of amino acids in the open reading frame (ORF), molecular weight (MW), isoelectric point (pI), and length of the open reading frame (length) for each gene [45].

### 4.2. PeUBP Gene Alignments and Phylogenetic Analysis

Four data sets were used for the phylogenetic analysis. In total, 27 Arabidopsis UBP protein sequences were downloaded from TAIR (https://www.arabidopsis.org/), and 25 rice UBP protein sequences and 32 Brachypodium UBP protein sequences were downloaded from the Phytozome (https://phytozome.jgi.doe.gov/pz/portal.html) to use as references. A multiple alignment of all the sequences was performed using W algorithm integration [52]. The Neighbor-joining method was used to construct a phylogenetic tree by MEGA 6.0 with bootstrap values calculated using 1000 replicates, and p-distance methods were used with the pairwise deletion option to address gaps in the amino acid sequences [53]. 

### 4.3. Gene Structure Analysis

The intron-exon structures of each gene were mapped to their corresponding genomic sequences. The intron-exon structures were determined by comparing CDSs with their corresponding genomic DNA sequences, and schematics were generated using the GSDS v2.0 (http://gsds.cbi.pku.edu.cn/) [54].

### 4.4. Protein Structure Analyses

We used the Phyre2 website (http://www.sbg.bio.ic.ac.uk/phyre2/html/page.cgi?id=index) to predict protein homology models, and applied Hidden Markov Models (HMM) to the alignment of the UBP protein sequences using the HMM–HMM search in intensive mode [55]. Conserved motifs present in the PeUBP proteins were identified with the online MEME tool (http://meme-suite.org/tools/meme), with the following parameter settings: maximum number of motifs—10, and maximum width—50. 

### 4.5. Calculation of Ka/Ks Values

Ka/Ks values mean the ratio of the number of nonsynonymous substitutions per nonsynonymous site (Ka) to the number of synonymous substitutions per synonymous site (Ks). Ka and Ks were calculated using KaKs Calculator 2.0 with the NY model [56] based on the pairwise alignment of paralogous and orthologous pairs between moso bamboo, and both rice and *Brachypodium*. Clustal W 2.1 was used to study the gene duplication events. The divergence time (T) was calculated according to T = Ks/2 λ (λ = 6.5 × 10^−9^) by converting the date of duplication events for moso bamboo, rice, and *Brachypodium* [24]. 

### 4.6. Putative Promoter Region Analysis of PeUBP Genes

The 2000-bp upstream sequences of the PeUBP genes were chosen to identify the cis-elements in the putative promoter regions. We used the PlantCARE (http://www.dna.affrc.go.jp/PLACE/) to predict the putative cis-regulatory elements present in the promoter sequences [57,58]. Then, we screened out cis-elements that responded to ABA, MeJA, and SA stress. 

### 4.7. Plant Material and Growth Conditions

THE 90-d-old moso bamboo seeds (from Gongcheng Yao Autonomous County, Guangxi Zhuang Autonomous Region, China) were germinated and grown in an artificial growth chamber under long daylight conditions (16-h light/8-h dark) maintained at 28 °C and 80% relative humidity. These seedlings were used in experiments to analyze gene expression levels in response to three stress treatments when they were 3 months of age. To study the expression patterns of PeUBP genes under stress-related hormone treatments, the young leaves were sprayed individually with 20 μM ABA, 100 μM MeJA, or 1 mM SA. The young leaves sprayed with distilled water were used as negative controls. The leaves of the hormone-treated plants were collected at 0, 3, 6, 12, and 24 h and all the samples were rapidly frozen in liquid nitrogen and stored at −80 °C prior to RNA extraction.

### 4.8. PeUBP Expression Levels in Different Tissues

The samples (leaf, root, stem, and rhizome) were collected from a 90-d-old moso bamboo. Leaf, root, stem, and rhizome refer to the young leaves, the first three tender stems, stems with roots in the last two segments, and roots growing in the soil, respectively. Then, we determined a comprehensive expression profile for each PeUBP genes, using the *Tonoplast intrinsic protein 41* (*TIP41*) gene as an internal standard [59,60]. The heatmap of PeUBP gene expression was constructed using R studio for four moso bamboo tissues (leaf, root, stem, and rhizome).

### 4.9. RNA Isolation and qRT-PCR

The qRT-PCR was performed using 48 genes based on their similarity levels to the reference genes in the phylogenetic tree. The primers of qRT-PCR of the 48 PeUBP genes are listed in Table S4. Total RNA was isolated from samples of each tissue using an RNAprep Pure Plant Kit (TransGen Biotech, Beijing, China) according to the user manual. Total RNAs were used for complementary cDNA synthesis using SuperScript III transcriptase (Invitrogen, Carlsbad, American) in accordance with the manufacturer’s instructions [61]. The qRT-PCR analysis was performed on a Bio-Rad CFX96 using the Light Cycler 480 SYBR Green Master Mix (TaKaRa, Dalian, China). The PCR reaction conditions were as follows: 95 °C for 30 s, followed by 40 cycles of 95 °C for 5 s, and 60 °C for 30 s. The expression values of the individual genes were normalized using the expression level of *TIP41* as an internal standard [60]. The mean expression values and SE values were calculated from the results of three independent experiments.

## 5. Conclusions

In this study, 48 UBP family genes in moso bamboo were identified and characterized by the systematic analyses of a phylogenetic tree, protein structure, gene structure, structural domains, and divergence time, which indicated a complex evolutionary history for this family in moso bamboo. The expression profiles of UBP genes indicated that these genes play pivotal roles in stress responses, as well as growth and development. This is a comprehensive study of moso bamboo UBP genes that may aid in the selection of appropriate candidate genes for further cloning and functional analyses in moso bamboo.

## Figures and Tables

**Table 1:** Detailed information about 48 predicted PeUBP proteins in moso bamboo. The last column lists the number of exons in each gene. CDS, the coding sequence of a gene; MW, molecular weight; pI, protein isoelectric point.

Gene ID	Location	CDS Length (bp)	Size (aa)	Protein	pl	Exons
				MW (Da)		
PH02Gene00721.t1	16: 30179141-30199136	3333	1110	129890.52	5.57	32
PH02Gene01291.t1	18: 35966647-35977509	2721	906	99762.48	6.22	12
PH02Gene02188.t1	24:40155854-40161467	1107	368	41908.50	5.88	6
PH02Gene02290.t2	23:13745085-13751574	1896	631	69325.70	4.85	13
PH02Gene02699.t1	6:13203375-13209603	2823	940	102597.05	9.36	11
PH02Gene03813.t1	3:84811131-84816494	2223	740	82069.42	4.92	15
PH02Gene05436.t1	15:67480992-67493851	3228	1075	119371.13	6.08	13
PH02Gene05450.t1	15:67882508-67890349	1101	366	42022.55	5.86	6
PH02Gene06421.t1	7:52268122-52288354	3354	1117	131095.16	5.62	32
PH02Gene08309.t1	4:54406565-54413534	2673	890	99416.65	5.55	12
PH02Gene08485.t1	9:53943713-53950726	1971	656	71789.57	8.98	8
PH02Gene09038.t1	16:113303229-113317987	2383	795	88955.33	5.27	19
PH02Gene11139.t1	8:10275805-10281089	2805	934	101955.24	9.34	11
PH02Gene11290.t1	1:21490093-21516076	3354	1117	131044.10	5.59	32
PH02Gene12835.t1	14:60797306-60818319	3249	1082	126562.99	5.71	30
PH02Gene13480.t1	10:1249930-1254111	1662	553	60007.42	8.70	7
PH02Gene15253.t1	21:101647723-101655588	2748	915	101640.25	6.34	10
PH02Gene15270.t1	21:102143034-102149220	1101	366	41960.49	6.13	6
PH02Gene15962.t1	13:51305905-51310945	2547	848	93,234.54	4.94	4
PH02Gene16195.t2	14:31330299-31338713	1425	474	53,211.28	5.87	17
PH02Gene18065.t1	11:42415269-42423636	2643	880	98195.73	5.11	13
PH02Gene19598.t1	3:10292006-10297292	3147	1048	113926.14	5.19	2
PH02Gene21213.t1	3:99405737-99411540	2979	992	109834.65	8.62	12
PH02Gene21515.t1	12:47774473-47795019	2694	897	100426.26	5.37	12
PH02Gene22284.t2	10:28962334-28980176	3429	1142	134013.04	5.66	31
PH02Gene22492.t1	6:34432327-34438439	2907	968	106038.69	5.96	11
PH02Gene24230.t1	2835:25003-30357	2217	738	81849.15	5.09	15
PH02Gene25343.t1	16:69739339-69750041	1425	474	53184.22	6.01	17
PH02Gene26126.t1	4:53402592-53419429	3360	1119	131489.97	5.71	32
PH02Gene28362.t1	23:7647625-7650488	1200	399	45414.32	7.91	3
PH02Gene28512.t1	13:58209169-58217387	2439	812	89586.32	6.69	10
PH02Gene29714.t1	20:29015633-29030162	2889	962	107226.58	7.13	13
PH02Gene30769.t1	18:30083038-30088423	3111	1036	113047.92	5.11	2
PH02Gene31615.t1	17:78162911-78172473	2673	890	98449.22	6.41	14
PH02Gene33187.t1	13:102796122-102807090	2808	935	105880.58	5.04	13
PH02Gene33419.t1	12:32620099-32636788	3357	1118	131431.81	5.71	32
PH02Gene33483.t1	17:40198974-40203749	1230	409	46321.46	5.85	6
PH02Gene33959.t1	5:54814061-54820578	2469	822	92602.24	5.49	14
PH02Gene35679.t1	4:3711703-3715914	1662	553	60216.88	8.88	7
PH02Gene37010.t1	18:22571046-22581812	2004	667	73289.87	9.21	3
PH02Gene39804.t1	5:3099809-3109660	2808	935	105866.37	5.02	13
PH02Gene42176.t1	8:66644923-66652376	3180	1059	115732.16	5.90	11
PH02Gene42469.t1	23:34433041-34449409	1107	368	41893.49	5.98	6
PH02Gene43669.t1	14:97907481-97915260	1962	733	81520.86	4.99	17
PH02Gene43803.t1	7:39435913-39442930	1961	653	71613.43	8.84	8
PH02Gene46815.t1	1:15050591-15059698	3810	1269	144256.52	6.22	3
PH02Gene47007.t1	1:15242214-15246894	3693	1320	151083.45	5.89	3
PH02Gene48223.t1	13:32005753-32013346	2565	855	96768.17	5.70	12

**Table 2:** Paralogous (Pe-Pe) and orthologous (Pe-Os and Pe-Bd) gene pairs.

Pe-Pe	Pe-Os	Pe-Bd
PH02Gene05450.t1/PH02Gene15270.t1	PH02Gene09038.t1/Os01g08200	PH02Gene05436.t1/Bradi1g07717
PH02Gene11139.t1/PH02Gene02699.t1	PH02Gene16195.t2/Os01g36930	PH02Gene13480.t1/Bradi1g18790
PH02Gene11290.t1/PH02Gene06421.t1	PH02Gene00721.t1/Os01g56490	PH02Gene11139.t1/Bradi1g47320
PH02Gene12835.t1/PH02Gene00721.t1	PH02Gene29714.t1/Os02g14730	PH02Gene26126.t1/Bradi1g56780
PH02Gene15253.t1/PH02Gene05436.t1	PH02Gene33483.t1/Os02g36400	PH02Gene08309.t1/Bradi1g57080
PH02Gene21213.t1/PH02Gene11139.t1	PH02Gene05450.t1/Os03g09080	PH02Gene05450.t1/Bradi1g71830
PH02Gene21515.t1/PH02Gene18065.t1	PH02Gene42469.t1/Os04g37950	PH02Gene09038.t1/Bradi2g04830
PH02Gene24230.t1/PH02Gene03813.t1	PH02Gene43803.t1/Os05g43480	PH02Gene47007.t1/Bradi2g14560
PH02Gene25343.t1/PH02Gene16195.t2	PH02Gene42176.t1/Os06g44380	PH02Gene43803.t1/Bradi2g20280
PH02Gene26126.t1/PH02Gene22284.t2	PH02Gene08309.t1/Os07g06610	PH02Gene16195.t2/Bradi2g40820
PH02Gene28512.t1/PH02Gene01291.t1	PH02Gene28512.t1/Os08g37350	PH02Gene00721.t1/Bradi2g51255
PH02Gene30769.t1/PH02Gene19598.t1	PH02Gene15962.t1/Os08g41540	PH02Gene39804.t1/Bradi3g20790
PH02Gene31615.t1/PH02Gene29714.t1	PH02Gene01291.t1/Os09g28940	PH02Gene33959.t1/Bradi3g34176
PH02Gene35679.t1/PH02Gene13480.t1	PH02Gene30769.t1/Os09g32740	PH02Gene28512.t1/Bradi3g38400
PH02Gene37010.t1/PH02Gene15962.t1	PH02Gene33187.t1/Os10g07270	PH02Gene15962.t1/Bradi3g41010
PH02Gene39804.t1/PH02Gene33187.t1	PH02Gene06421.t1/Os11g36470	PH02Gene33483.t1/Bradi3g46610
PH02Gene42176.t1/PH02Gene22492.t1	PH02Gene33419.t1/Os12g30540	PH02Gene24230.t1/Bradi3g52210
PH02Gene42469.t1/PH02Gene02188.t1	PH02Gene18065.t1/Os12g42600	PH02Gene18065.t1/Bradi4g01310
PH02Gene43669.t1/PH02Gene09038.t1		PH02Gene11290.t1/Bradi4g15710
PH02Gene43803.t1/PH02Gene08485.t1		PH02Gene48223.t1/Bradi4g19177
PH02Gene47007.t1/PH02Gene46815.t1		PH02Gene42469.t1/Bradi5g12000
		PH02Gene28362.t1/Bradi5g23920

## Supplementary Materials

Supplementary materials can be found at https://www.mdpi.com/1422-0067/20/17/4309/s1.

## Abbreviations

UBPs	Ubiquitin-specific proteases
DUBs	Deubiquitinases/Deubiquitinating enzymes
ABA	Abscisic acid
MeJA	Methyl jasmonate
SA	Salicylic acid
E1	Ubiquitin activating enzyme
E2	Ubiquitin conjugating enzyme
E3	Ubiquitin ligase=
UCH	Carboxyl-terminal hydrolase
Cys	Cysteine
His	Histidine
DUSP	Domain present in UBP
