# Supplementary material for: Genome-Wide Identification and Characterization of the UBP Gene Family in Moso Bamboo (Phyllostachys edulis)
Source: Int J Mol Sci. 2019 Sep 3;20(17):4309. doi: 10.3390/ijms20174309 (PMC6747111; doi:10.3390/ijms20174309)
Supplement: Supplementary file 1 [file ijms-20-04309-s001.zip › Supplementary/Table S1-Detailed information about UBP genes in Arabidopsis thaliana, Oryza sativa and Brachypodium distachyon..docx]

Table S1. Detailed information about UBP genes in *Arabidopsis thaliana, Oryza sativa* and *Brachypodium distachyon*.

| **Sequences ID** | **Mol.Wt.(KDa)** | **PI** | **Length of CDS** | **Size(aa)** |
| --- | --- | --- | --- | --- |
| AT2G32780 | 120770.70 | 4.84 | 3252 | 1083 |
| AT1G04860 | 105152.35 | 5.14 | 2886 | 961 |
| AT4G39910 | 42447.14 | 6.08 | 1116 | 371 |
| AT2G22310 | 37863.14 | 6.17 | 987 | 328 |
| AT2G40930 | 103874.91 | 5.52 | 2775 | 924 |
| AT1G51710 | 53695.90 | 5.82 | 1449 | 482 |
| AT3G21280 | 59832.16 | 5.97 | 1599 | 532 |
| AT5G22030 | 98170.96 | 5.20 | 2742 | 871 |
| AT4G10570 | 103698.56 | 5.12 | 2772 | 923 |
| AT4G10590 | 102649.40 | 5.06 | 2733 | 910 |
| AT1G32850 | 101175.16 | 5.48 | 2679 | 892 |
| AT5G06600 | 130606.95 | 5.51 | 3351 | 1116 |
| AT3G11910 | 130648.82 | 5.38 | 3348 | 1115 |
| AT3G20630 | 88373.84 | 5.05 | 2394 | 797 |
| AT1G17110 | 103676.50 | 8.01 | 2787 | 924 |
| AT4G24560 | 110600.32 | 6.73 | 3027 | 1008 |
| AT5G65450 | 81998.98 | 8.60 | 2196 | 731 |
| AT4G31670 | 70752.59 | 4.97 | 1896 | 631 |
| AT2G24640 | 75719.55 | 4.98 | 2019 | 672 |
| AT4G17895 | 78632.91 | 4.45 | 2088 | 695 |
| AT5G46740 | 82417.13 | 5.97 | 2199 | 732 |
| AT5G10790 | 63517.52 | 8.03 | 1674 | 557 |
| AT5G57990 | 94903.11 | 9.31 | 2580 | 859 |
| AT4G30890 | 60440.05 | 7.70 | 1656 | 551 |
| AT3G14400 | 73293.14 | 9.21 | 1986 | 661 |
| AT3G49600 | 117566.12 | 5.73 | 3204 | 1046 |
| AT4G39370 | 57035.26 | 6.29 | 1518 | 505 |
| LOC_Os01g08200 | 88664.89 | 5.08 | 2385 | 794 |
| LOC_Os01g36930 | 53165.12 | 5.79 | 1425 | 474 |
| LOC_Os01g56490 | 129096.93 | 5.42 | 3333 | 1110 |
| LOC_Os02g14730 | 100233.01 | 6.91 | 2724 | 907 |
| LOC_Os02g36400 | 46533.51 | 6.12 | 1248 | 415 |
| LOC_Os02g55180 | 129213.97 | 7.72 | 2352 | 1185 |
| LOC_Os03g09080 | 42027.74 | 5.80 | 1101 | 366 |
| LOC_Os04g34984 | 59319.28 | 6.04 | 1617 | 538 |
| LOC_Os04g37950 | 41793.46 | 6.09 | 1104 | 367 |
| LOC_Os05g43480 | 70727.51 | 9.12 | 1935 | 644 |
| LOC_Os06g08530 | 102958.13 | 9.45 | 2748 | 940 |
| LOC_Os06g44380 | 115914.97 | 5.76 | 3210 | 1069 |
| LOC_Os07g06610 | 98972.93 | 5.33 | 2658 | 885 |
| LOC_Os07g46660 | 60655.65 | 7.67 | 1668 | 558 |
| LOC_Os08g37350 | 107730.92 | 8.26 | 2937 | 978 |
| LOC_Os08g41530 | 105686.86 | 5.23 | 2880 | 959 |
| LOC_Os08g41540 | 96075.98 | 5.11 | 2610 | 869 |
| LOC_Os08g41620 | 82821.66 | 5.51 | 2253 | 750 |
| LOC_Os08g41630 | 105072.86 | 5.55 | 2841 | 946 |
| LOC_Os09g28940 | 103188.66 | 6.64 | 2814 | 937 |
| LOC_Os09g32740 | 113992.85 | 4.98 | 3168 | 1055 |
| LOC_Os10g07270 | 105050.38 | 5.02 | 2796 | 931 |
| LOC_Os11g36470 | 110627.56 | 5.81 | 2817 | 938 |
| LOC_Os12g30540 | 131937.27 | 5.54 | 3378 | 1125 |
| LOC_Os12g42600 | 89131.47 | 5.63 | 2406 | 801 |
| Bradi1g07717 | 119433.42 | 6.00 | 3225 | 1074 |
| Bradi1g18790 | 79005.73 | 8.57 | 2232 | 742 |
| Bradi1g31187 | 114145.33 | 6.05 | 3141 | 1048 |
| Bradi1g47320 | 102375.58 | 9.31 | 2817 | 938 |
| Bradi1g56780 | 131339.61 | 5.56 | 3369 | 1122 |
| Bradi1g57080 | 98015.11 | 5.40 | 2649 | 882 |
| Bradi1g71830 | 41932.40 | 5.86 | 1101 | 366 |
| Bradi2g04830 | 88736.91 | 5.06 | 2502 | 795 |
| Bradi2g14560 | 149775.66 | 6.46 | 3924 | 1307 |
| Bradi2g20280 | 70729.63 | 8.84 | 1926 | 641 |
| Bradi2g40820 | 47933.03 | 5.69 | 1578 | 429 |
| Bradi2g51255 | 126968.54 | 5.82 | 3258 | 1085 |
| Bradi3g09260 | 103999.90 | 6.88 | 2916 | 940 |
| Bradi3g20790 | 105194.60 | 5.18 | 2796 | 931 |
| Bradi3g34174 | 53171.03 | 4.72 | 1479 | 467 |
| Bradi3g34176 | 81731.26 | 6.79 | 2148 | 715 |
| Bradi3g38400 | 106745.44 | 7.35 | 2913 | 970 |
| Bradi3g40994 | 114064.74 | 5.80 | 3123 | 1040 |
| Bradi3g40998 | 105028.08 | 4.95 | 2877 | 958 |
| Bradi3g41010 | 95983.18 | 4.76 | 2631 | 876 |
| Bradi3g46610 | 46251.35 | 5.94 | 1230 | 409 |
| Bradi3g52210 | 81468.52 | 5.13 | 2205 | 734 |
| Bradi3g54040 | 116583.57 | 7.66 | 2952 | 1051 |
| Bradi4g01310 | 99774.52 | 5.53 | 2703 | 900 |
| Bradi4g07167 | 131397.74 | 5.59 | 3360 | 1118 |
| Bradi4g15430 | 141865.67 | 6.65 | 3744 | 1247 |
| Bradi4g15710 | 131060.15 | 5.58 | 3360 | 1119 |
| Bradi4g19177 | 103664.20 | 5.92 | 2751 | 916 |
| Bradi4g32500 | 75308.17 | 6.14 | 2949 | 685 |
| Bradi5g10117 | 59291.29 | 6.49 | 1611 | 535 |
| Bradi5g12000 | 41797.34 | 5.98 | 1107 | 368 |
| Bradi5g23920 | 62681.91 | 8.70 | 1692 | 563 |
