# Supplementary material for: Genome-Wide Identification and Characterization of the UBP Gene Family in Moso Bamboo (Phyllostachys edulis)
Source: Int J Mol Sci. 2019 Sep 3;20(17):4309. doi: 10.3390/ijms20174309 (PMC6747111; doi:10.3390/ijms20174309)
Supplement: Supplementary file 1 [file ijms-20-04309-s001.zip › Supplementary/Table S3-The microarray data of 48 PeUBP genes in moso bamboo..docx]

Table S3. The microarray data of 48 PeUBP genes in moso bamboo.

| Name | leaf | stem | rhizome | root |
| --- | --- | --- | --- | --- |
| PH02Gene00721.t1 | 1.12354 | 0.75424 | 0.8522 | 0.94525 |
| PH02Gene01291.t1 | 1.2355 | 0.82345 | 0.72134 | 0.7425 |
| PH02Gene02188.t1 | 1.17561 | 0.97376 | 0.84405 | 1.6418 |
| PH02Gene02290.t2 | 0.94536 | 0.64324 | 0.42412 | 0.68234 |
| PH02Gene02699.t1 | 0.952367 | 0.79342 | 0.63412 | 0.89232 |
| PH02Gene03813.t1 | 0.89624 | 0.62743 | 0.68422 | 1.09352 |
| PH02Gene05436.t1 | 0.90934 | 0.29098 | 0.20182 | 0.87926 |
| PH02Gene05450.t1 | 1.20674 | 0.42555 | 0.635423 | 0.635211 |
| PH02Gene06421.t1 | 1.10439 | 0.38367 | 0.456372 | 0.52134 |
| PH02Gene08309.t1 | 1.18543 | 0.39232 | 0.59863 | 0.93833 |
| PH02Gene08485.t1 | 0.98736 | 0.369785 | 0.24556 | 0.75443 |
| PH02Gene09038.t1 | 0.96624 | 0.41231 | 0.36744 | 0.57853 |
| PH02Gene11139.t1 | 1.09784 | 0.63422 | 0.98632 | 0.6092 |
| PH02Gene11290.t1 | 1.07543 | 0.93432 | 0.5342 | 0.457542 |
| PH02Gene12835.t1 | 1.02124 | 0.57343 | 0.3743 | 0.63453 |
| PH02Gene13480.t1 | 1.0671 | 1 | 0.21995 | 0.52255 |
| PH02Gene15253.t1 | 0.91234 | 0.8352 | 0.567452 | 0.45647 |
| PH02Gene15270.t1 | 0.98234 | 0.267456 | 0.74535 | 0.8453 |
| PH02Gene15962.t1 | 0.89874 | 0.6322 | 0.56832 | 0.42135 |
| PH02Gene16195.t2 | 0.92123 | 0.75321 | 0.64231 | 0.57452 |
| PH02Gene18065.t1 | 0.95432 | 0.32463 | 0.34575 | 0.47542 |
| PH02Gene19598.t1 | 0.93738 | 0.20187 | 0.55362 | 0.55737 |
| PH02Gene21213.t1 | 0.91685 | 1.2241 | 0.86778 | 0.87884 |
| PH02Gene21515.t1 | 1.03234 | 0.8531 | 0.34562 | 0.47531 |
| PH02Gene22284.t2 | 1.09843 | 0.82421 | 0.27653 | 0.43125 |
| PH02Gene22492.t1 | 1.03654 | 0.47952 | 0.84421 | 0.31754 |
| PH02Gene24230.t1 | 1.104354 | 0.567682 | 0.74256 | 0.742135 |
| PH02Gene25343.t1 | 1.05737 | 0.96531 | 0.8817 | 0.62684 |
| PH02Gene26126.t1 | 0.89245 | 0.35323 | 0.72346 | 0.387545 |
| PH02Gene28362.t1 | 0.984253 | 0.55324 | 0.68533 | 0.452321 |
| PH02Gene28512.t1 | 0.81217 | 0.33753 | 0.1683 | 0.60084 |
| PH02Gene29714.t1 | 1.10321 | 0.26756 | 0.321675 | 0.48655 |
| PH02Gene30769.t1 | 0.98421 | 0.7342 | 0.16424 | 0.375345 |
| PH02Gene31615.t1 | 1.104232 | 0.424745 | 0.57435 | 0.37456 |
| PH02Gene33187.t1 | 1.09422 | 0.76435 | 0.53567 | 0.58792 |
| PH02Gene33419.t1 | 0.98761 | 0.8856 | 0.74641 | 0.9839 |
| PH02Gene33483.t1 | 0.99345 | 0.80332 | 0.87877 | 0.62237 |
| PH02Gene33959.t1 | 0.80955 | 0.61488 | 0.75428 | 1.26326 |
| PH02Gene35679.t1 | 0.89422 | 0.345365 | 0.578644 | 1.100543 |
| PH02Gene37010.t1 | 0.789342 | 0.63556 | 0.97453 | 0.76333 |
| PH02Gene39804.t1 | 0.91234 | 0.6536 | 0.8563 | 1.04216 |
| PH02Gene42176.t1 | 1.09324 | 0.567433 | 0.9435 | 1.26377 |
| PH02Gene42469.t1 | 1.10854 | 0.3636 | 0.645422 | 1.04236 |
| PH02Gene43669.t1 | 1.15175 | 1 | 0.77948 | 0.79346 |
| PH02Gene43803.t1 | 1 | 0.14522 | 0.24629 | 0.0265 |
| PH02Gene46815.t1 | 0.95498 | 0.70896 | 0.7775 | 0.82308 |
| PH02Gene47007.t1 | 0.98245 | 0.68953 | 0.47478 | 0.95632 |
| PH02Gene48223.t1 | 0.70956 | 0.74214 | 1.06883 | 1.12172 |
