# Supplementary material for: Genome-Wide Identification and Characterization of the UBP Gene Family in Moso Bamboo (Phyllostachys edulis)
Source: Int J Mol Sci. 2019 Sep 3;20(17):4309. doi: 10.3390/ijms20174309 (PMC6747111; doi:10.3390/ijms20174309)
Supplement: Supplementary file 1 [file ijms-20-04309-s001.zip › Supplementary/Table S4-The primers of qRT-PCR of 48 PeUBP genes..docx]

Table S4. The primers of qRT-PCR of 48 PeUBP genes.

| **Genes** | **Primers used in qRT-PCR(5′→3′)** | |
| --- | --- | --- |
| PH02Gene00721.t1 | Forwards | ACGATTGCCGAAAGGCAGTA |
|  | Reverse | GGGAACCTCTTCAACTCGCA |
| PH02Gene01291.t1 | Forwards | AGTCGTATGAGCGTGCCAAA |
|  | Reverse | GAGCCTGTACACAGGGGAAC |
| PH02Gene02188.t1 | Forwards | GGCCCCAACCATGGACATTA |
|  | Reverse | CCACCAAGGCCCTCGTAAAA |
| PH02Gene02290.t2 | Forwards | CGGAGACTCTGAGGACAAGC |
|  | Reverse | CTGGGTTGCTGTGAAATGGC |
| PH02Gene02699.t1 | Forwards | AAGGCCACTACGTTGGTGAG |
|  | Reverse | TTTCACGAGGGTCGAAAGGG |
| PH02Gene03813.t1 | Forwards | TGCACCCTTGATCCCAAGTC |
|  | Reverse | TCCAAAGCACCAACGAGGTT |
| PH02Gene05436.t1 | Forwards | GTCGCACTTAACCCAGGGAA |
|  | Reverse | CGCGCTTGCAAACTAGATCC |
| PH02Gene05450.t1 | Forwards | ATAAAAGCGCCGGTGATGGT |
|  | Reverse | TCCTGGTGCATATAGCTGCG |
| PH02Gene06421.t1 | Forwards | ACGATCGAGAACTTCACCCG |
|  | Reverse | AACGGCTCCAACCATAAGGG |
| PH02Gene08309.t1 | Forwards | GACTGTGCTGCTGACCTGAT |
|  | Reverse | TGCTGGACATCACCCCTTTC |
| PH02Gene08485.t1 | Forwards | ACCAGTGATGCTAAGGCCAC |
|  | Reverse | TCAAGAGGGCGTTTCAAGCA |
| PH02Gene09038.t1 | Forwards | TTGTTGAGGAGCGGGTTCAA |
|  | Reverse | TGGGACTCTAGGCCTCACAA |
| PH02Gene11139.t1 | Forwards | ACGAGCAGCAGACATCAACA |
|  | Reverse | AGCTCGGCACTAGCAAAGTT |
| PH02Gene11290.t1 | Forwards | GTTCACGTGGACGATCGAGA |
|  | Reverse | AACGGCTCCAACCATAAGGG |
| PH02Gene12835.t1 | Forwards | GTCAAGGGCTGCTCAGATGT |
|  | Reverse | CGGGAACTCGTAACGGTCAT |
| PH02Gene13480.t1 | Forwards | AGATCCAGTTTGGGTCGCTG |
|  | Reverse | CGGCCCACCATTTGTTACAC |
| PH02Gene15253.t1 | Forwards | CCATGCGAAATCTGCAGCAA |
|  | Reverse | TCGCGGTAACGTAAGCTCTC |
| PH02Gene15270.t1 | Forwards | AAAAGTGCCGGAGATGGTGA |
|  | Reverse | TGGTGCATATAGCTGCGGAA |
| PH02Gene15962.t1 | Forwards | GGACTGCCTGGCACTGTATT |
|  | Reverse | TGCCAAGCTGTTCAAATCGC |
| PH02Gene16195.t2 | Forwards | AAGAAGAGCAAGTGGTCGCA |
|  | Reverse | AGTGTAGAGGTGCAACTGGC |
| PH02Gene18065.t1 | Forwards | TCTGCCAGGGCCAATACAAG |
|  | Reverse | TGTAAGGAGATGGCCCGGTA |
| PH02Gene19598.t1 | Forwards | GTCGTGAGCTGCAGCAAAAA |
|  | Reverse | TCCCTACACTCTTCGCGGTA |
| PH02Gene21213.t1 | Forwards | GAGGATAGAGTTCCACGCCG |
|  | Reverse | CTAAGCTCCCGATCGAACCC |
| PH02Gene21515.t1 | Forwards | TGCTGCAGAGTGCACCAATA |
|  | Reverse | TTGCTCGAGTCCATTGTGCT |
| PH02Gene22284.t2 | Forwards | CCAAGCGGCAAAACCATACC |
|  | Reverse | CAGCGGAAGAGGTTCAAGGT |
| PH02Gene22492.t1 | Forwards | AAGAAGTGTTCCGGGTGCAA |
|  | Reverse | CCATGGGCAGGCACTAATGA |
| PH02Gene24230.t1 | Forwards | GTTCTAAGGCCAGCGAGGTT |
|  | Reverse | CACACTCGCAGCAAGTCAAC |
| PH02Gene25343.t1 | Forwards | CAGGGACAAAGCCGATTCCT |
|  | Reverse | CCAGTCCCCTCCACCAGATA |
| PH02Gene26126.t1 | Forwards | CCCGATGCAGACAGGAATGT |
|  | Reverse | TGAGGTAGCTCCTCCTCACC |
| PH02Gene28362.t1 | Forwards | GACGAGATCTACTCTGCCGC |
|  | Reverse | GGCCTTGGGCATGTGATTTG |
| PH02Gene28512.t1 | Forwards | GCAAGGAGGAGGTTCGTTGA |
|  | Reverse | GCTTTCGCTGCTATCTGTGC |
| PH02Gene29714.t1 | Forwards | TGCAGACTCGGTTAGCACAG |
|  | Reverse | TGGATTGGAAGGCGAGAACC |
| PH02Gene30769.t1 | Forwards | ACTGAAGCGCGAAAGCTAGT |
|  | Reverse | TCGCTGGTGGTGATGAAACA |
| PH02Gene31615.t1 | Forwards | ACGTCAAGTGAATGGTCGCT |
|  | Reverse | TGGATTGGAAGGCGAGAACC |
| PH02Gene33187.t1 | Forwards | GGAAGCTCATTGGAAGCCCT |
|  | Reverse | TTCTCAGTGGAAGCGCCATT |
| PH02Gene33419.t1 | Forwards | ATGGAGCGCGTTCAGAGAAA |
|  | Reverse | TCCCAAGCTCCGTAAACGTC |
| PH02Gene33483.t1 | Forwards | CCAGGGACACTACGTAAGCC |
|  | Reverse | CTTCCCACCAAGGCACTCAT |
| PH02Gene33959.t1 | Forwards | GAGGAGTCCACGTCTTGTGG |
|  | Reverse | AACCCGCCAACATACCCAAT |
| PH02Gene35679.t1 | Forwards | GCCAAGGCAAGAAGATGCTC |
|  | Reverse | ACCTGTAGCTTTCACGAGGC |
| PH02Gene37010.t1 | Forwards | GTCAAGGAAGACGCCGGTAA |
|  | Reverse | CCAACCAATGCTGCACCTTC |
| PH02Gene39804.t1 | Forwards | GGAAGCTCATTGGAAGCCCT |
|  | Reverse | GGCGCCATTATCCGTCAGTA |
| PH02Gene42176.t1 | Forwards | TGGCAACCTTGGTTTCGGTA |
|  | Reverse | GAGATTGCAGACGACCACCA |
| PH02Gene42469.t1 | Forwards | GGCCCCAACCATGGACATTA |
|  | Reverse | CCACCAAGGCCCTCGTAAAA |
| PH02Gene43669.t1 | Forwards | TTGTTGAGGAGCGGGTTCAA |
|  | Reverse | TGGGACTCTATGCCTCACGA |
| PH02Gene43803.t1 | Forwards | TGTTCCCGAACAGGGACAAG |
|  | Reverse | AAGCGTCTATGACGTAGCGG |
| PH02Gene46815.t1 | Forwards | TCTCGTCACACTGCTGTTCC |
|  | Reverse | CAGCGGAAACTGACAAGCAC |
| PH02Gene47007.t1 | Forwards | AAACCGTGAAGGATGCTCGT |
|  | Reverse | ATCGATCAGTTCCCTCCCCA |
| PH02Gene48223.t1 | Forwards | TGGTGGGTGAACTTGCGTTA |
|  | Reverse | ACAACTCCTGCGAATCGTGT |
| *PeTIP41* | Forwards | AAAATCATTGTAGGCCATTGTCG |
|  | Reverse | ACTAAATTAAGCCAGCGGGAGTG |
