# Supplementary figures and images for: Genome-Wide Identification and Characterization of the UBP Gene Family in Moso Bamboo (Phyllostachys edulis)
Source: Int J Mol Sci. 2019 Sep 3;20(17):4309. doi: 10.3390/ijms20174309 (PMC6747111; doi:10.3390/ijms20174309)

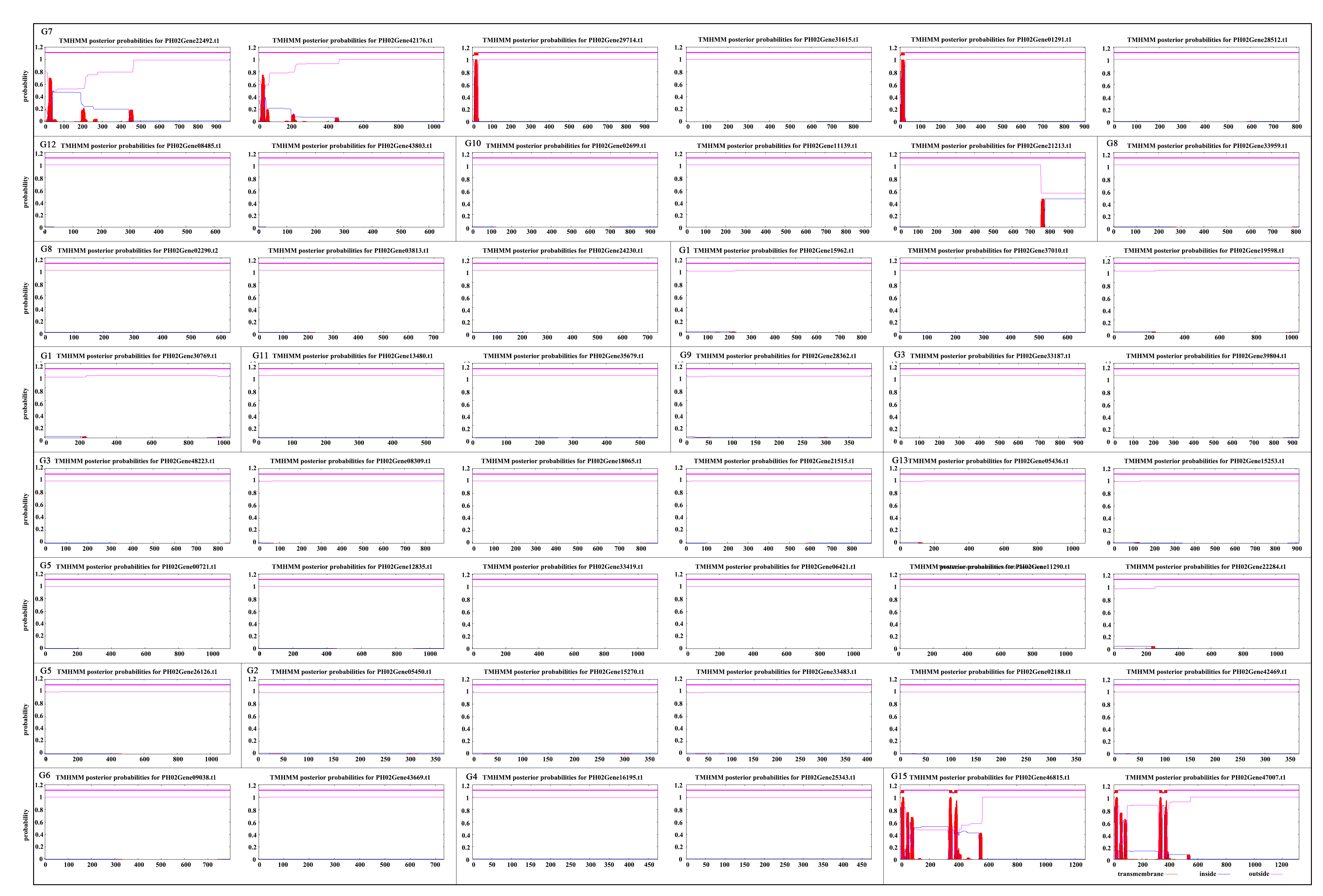

Supplement: Supplementary file 1 [file ijms-20-04309-s001.zip › Supplementary/Figure S1-The prediction of transmembrane (TM) regions of PeUBP proteins.png]
