# Supplementary material for: Genome-Wide Identification and Characterization of the UBP Gene Family in Moso Bamboo (Phyllostachys edulis)
Source: Int J Mol Sci. 2019 Sep 3;20(17):4309. doi: 10.3390/ijms20174309 (PMC6747111; doi:10.3390/ijms20174309)
Supplement: Supplementary file 1 [file ijms-20-04309-s001.zip › Supplementary/Table S2-The MEME motif sequences and lengths of PeUBP gene family proteins in moso bamboo.doc]

Table S2. The MEME motif sequences and lengths of PeUBP gene family proteins in moso bamboo.

| **Motif** | **Width** | **Best possible match** |
| --- | --- | --- |
| 1 | 23 | NLGNTCYMNSVLQCLYHTPPLRK |
| 2 | 29 | CKGLVDAKKQLLIKKAPPVLVIHLKRFSY |
| 3 | 41 | EETJIQQJFGGQLKSQVKCLRCGHVSVRFEPFMDLSLDIKG |
| 4 | 12 | GPNSGHYVAYVK |
| 5 | 21 | EDAHELLRFLLDKLHDALLKE |
| 6 | 50 | WYKFDDERVTKEDTKRALEEQYGGEEELPQTNPGLNNTPFKFTKYSNAYM |
| 7 | 33 | DSSGSWYRFDDSQVSPVSEEEVLSQKAYMLFYV |
| 8 | 21 | PNSDDDSSNLYSLYAVVVHSG |
| 9 | 29 | AKESGGPVAPKRFLSNLKRQNPQFRPGRQ |
| 10 | 50 | HMPTTENDMPSGSIPLALQSLFYKLQYSDSSVSTKELTKSFGWDTYDSFM |
